# Ursodeoxycholic acid attenuates the expression of proinflammatory cytokines in periodontal cells

**DOI:** 10.1002/JPER.19-0013

**Published:** 2020-02-06

**Authors:** Reza Talebian, Layla Panahipour, Reinhard Gruber

**Affiliations:** ^1^ Department of Oral Biology University Clinic of Dentistry, Medical University of Vienna Vienna Austria; ^2^ Experimental Research Center Medical Faculty Tehran University of Medical Sciences Tehran Iran; ^3^ Department of Periodontology School of Dental Medicine University of Bern Bern Switzerland

**Keywords:** in vitro, inflammation, liver cirrhosis, mouth, periodontitis, ursodeoxycholic acid

## Abstract

**Background:**

Ursodeoxycholic acid (UDCA) is one of the first‐line therapeutic medications used in treatment of cholestatic liver disease. Considering that periodontitis is epidemiologically linked to liver diseases, the question arises weather UDCA holds anti‐inflammatory properties on periodontal health. Herein, we provide information that support anti‐inflammatory effects of UDCA on three different periodontium‐related cell types.

**Methods:**

Gingival fibroblasts and the oral human squamous carcinoma cell line HSC‐2 were exposed to interleukin (IL)1β and tumor necrosis factor (TNF)α with and without UDCA. Murine RAW 264.7 macrophages were incubated with sterile‐filtered human saliva also in the presence of UDCA. The expression of inflammatory cytokines was measured by reverse transcription‐polymerase chain reaction. Immunoassay was applied to detect the production of IL6. Immunostaining was performed for the p65 subunit to further support the anti‐inflammatory role of UDCA.

**Results:**

We report here that UDCA significantly reduced the IL1β and TNFα‐induced expression of IL1, IL6, and IL8 in gingival fibroblasts and the HSC‐2 cell line. In RAW 264.7 macrophages, UDCA attenuated the expression of IL1α, IL1β, and IL6 that was increased by saliva. Immunoassay confirmed the capacity of UDCA to reduce inflammation‐induced production of IL6 in gingival fibroblasts, HSC‐2 and RAW 264.7 cells. Immunostaining revealed the blocking of nuclear translocation of p65 in gingival fibroblasts.

**Conclusions:**

Taken together, UDCA can attenuate the provoked expression of inflammatory cytokines in oral fibroblasts, oral human squamous carcinoma cells and macrophages in vitro. These data support the hypothesis that patients with cholestatic liver disease might benefit from UDCA with respect to periodontal health.

## 1 INTRODUCTION

Apart from plaque‐induced cellular responses that provoke an inflammatory reaction, also systemic diseases can be associated with the progression and the severity of periodontal disease.^1^ Epidemiologic evidences suggest that patients with scarring of the liver are at higher risk of periodontal diseases.^2^, ^3^ Risk factors for liver cirrhosis include alcohol abuse, being overweight or obese and viral hepatitis B or C, some of which are associated with the occurrence periodontal diseases.^4^ To date, there are no longitudinal studies proving that those diseases are a real risk factor. Apart from being associated with periodontal diseases, liver diseases constitute the third commonest cause of premature death in the U.K.^5^ and are recognized as the second leading cause of mortality amongst all digestive diseases in the United States.^6^ Thus, patients suffering from liver cirrhosis not only have a high mortality risk, they also require attention when it comes to diagnosis and treatment of periodontal diseases.

Patients with liver cirrhosis are frequently treated with the ursodeoxycholic acid (UDCA), also known as ursodiol.^7^, ^8^ UDCA is produced by intestinal bacteria also being the therapeutically active component of bear bile used in traditional Chinese medicine. UDCA can attenuate the symptoms of colitis induced by trinitrobenzene sulfonic acid in rat^9^ and dextran sodium sulphate model in mouse.^10^ These exemplary preclinical studies support a role of UDCA to exert anti‐inflammatory properties at the tissue level. In patients, UDCA improves transplant‐free survival in primary biliary cirrhosis. However, about 40% of patients do not respond to UDCA.^11^, ^12^ UDCA has been shown to be effective in non‐alcoholic steatohepatitis^13^ and the dissolution of cholesterol gallstones.^14^ UDCA may have a role to play in the therapy of inflammatory bowel diseases.^15^ Considering that patients with liver cirrhosis may suffer from periodontal diseases, they might benefit from the anti‐inflammatory properties of UDCA.

Further supports for the anti‐inflammatory function of UDCA come from in vitro studies as follows; UDCA can exert anti‐inflammatory effects in macrophages, for example, UDCA inhibits tumor necrosis factor (TNF)α‐induced release of interleukin (IL)8 from monocytes^16^ and lipopolysaccharide‐stimulated inflammatory responses in RAW 264.7 macrophages.^17^ Moreover, UDCA decreases the level of intracellular reactive oxidative species in pancreatic cancer cells.^18^ UDCA protects cardiomyocytes against hypoxia^19^ and attenuates symptoms in a rheumatoid arthritis mouse model.^20^ Mast cells treated with UDCA decreased histamine secretion.^21^ Therefore, accumulating evidence suggests anti‐inflammatory activity of UDCA at the tissue level and in vitro. However, if UDCA lowers the expression of inflammatory cytokines in oral fibroblasts and epithelial cells, as well as macrophages exposed to saliva^22^ remains unclear. The aim of this study was to determine the effect of UDCA on inflammatory cytokine expression on the oral fibroblasts, oral epithelial cells, and RAW 264.7 macrophages with the intention to show that the treatment of the cirrhosis could reduce the symptoms of periodontal disease, not that UDCA should be used for periodontal therapy.

## 2 MATERIALS AND METHODS

### 2.1 Human gingival fibroblasts and oral squamous carcinoma cells and HSC‐2 cells*Japanese Collection of Research Bioresources, Osaka, Japan.


Human gingival fibroblasts were harvested from extracted wisdom teeth from patients who had given written informed consent. This study was approved by the human subject's ethics board of Medical University of Vienna (EK NR 631/2007) and was conducted in accordance with the Helsinki Declaration of 1975, as revised in 2013. All methods were performed in accordance with the relevant guidelines and regulations. Two strains of fibroblasts were established and <10 passages were used for the experiments. The human oral squamous carcinoma cell line HSC‐2 and gingival fibroblasts were seeded at a concentration of 30,000 cells/cm² onto culture dishes 1 day before stimulation. Cells were exposed to IL1β and TNFα at the concentration of 5 ng/mL for 1 hour followed by the addition of UDCA†Sigma, St. Louis, MO. at 100 µM in serum‐free medium for 3 hours before RNA isolation. To harvest the supernatant, the experiment was extended to 24 hours.

### 2.2 RAW 264.7 and saliva preparation

For inflammatory experiments, RAW 264.7 cells‡American Type Culture Collection, Manassas, VA. were seeded at a concentration of 300,000 cells/cm² onto culture dishes 1 day before stimulation. Cells were exposed to 5% of fresh sterile saliva for 1 hour followed by the addition of 100 µM UDCA for 3 hours before preparing total RNA. To collect the supernatant, the experiment was prolonged for 24 hours. Whole human saliva was collected from the two authors (RT and RG) who were non‐smokers and gave their informed consent. Saliva flow was stimulated by chewing paraffin wax§Ivoclar Vivadent, Schaan, Liechtenstein. without eating and drinking for 1 hour before collection. Immediately after collection, saliva was centrifuged at 4,000 × g for 5 minutes. The supernatant was passed through a filter with a pore diameter of 0.2 µm.

### 2.3 Viability experiments

For cell viability, gingival fibroblasts were exposed to UDCA (62 µM − 2 mM) for 24 hours. Likewise, gingival fibroblasts, HSC‐2 and RAW 264.7 cells were stimulated with IL1β and TNFα at 5 ng/mL and saliva for 1 hour followed by the addition of UDCA at 100 µM in serum‐free medium for 3 hours. An MTT solution at a final concentration of 0.5 mg/mL was added to each well of a microtiter plate for 2 hours at 37°C. Medium was removed and formazan crystals were solubilized with dimethyl sulfoxide. Optical density was measured at 570 nm. Data were expressed as optical density in the treatment groups normalized to unstimulated control values. Also, gingival fibroblasts were exposed to 0.4% trypan blue for ≈1 minute to determine the integrity of the membrane.^23^

### 2.4 RT‐PCR and immunoassay

Reverse transcription (RT)*Bioline Reagents, London, U.K. and RT‐polymerase chain reaction (PCR)†LabConsulting, Vienna, Austria. were done with kits using manufacturer's instructions. Amplification was performed with a real‐time PCR device. The primers sequences are given in Supplemental Table S1 in online *Journal of Periodontology*. Relative gene expression was calculated based on GAPDH and β‐actin with the ΔΔCT method.‡Bio‐Rad Laboratories, Hercules, CA. Reactions were run in duplicates. The supernatant was analyzed for IL6 using an immunoassay according to the manufacturer's instructions.§R&D Systems, Minneapolis, MN.


### 2.5 Immunostaining

Immunofluorescence analysis was performed on gingival fibroblasts treated with IL1β and TNFα at a concentration of 5 ng/mL for 20 minutes before being exposed to 100 µM UDCA for 1 hour. Cells were fixed in paraformaldehyde and blocked in 1% BSA and 0.3% Triton in PBS for 1 hour at room temperature. Cells were subsequently incubated with nuclear factor (NF)kB p65 primary antibody¶Cell Signaling Technology Europe, Frankfurt am Main, Germany. and Alexa 488 secondary antibody#Santa Cruz Biotechnology, Dallas, TX. was applied for 1 hour. Cells were washed and mounted onto glass slides. Images were captured at 100× in oil immersion using a fluorescence microscope.

### 2.6 Statistical analysis

**Figure 1:**
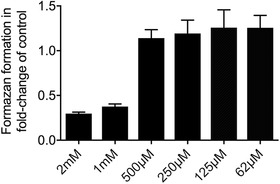
UDCA decreased the viability of oral fibroblasts at millimolar concentrations. Human gingival fibroblasts were exposed to increasing concentrations of UDCA for 24 hours before the production of formazan crystals was determined. Data show the production of formazan crystals normalized to untreated controls

**Figure 2:**
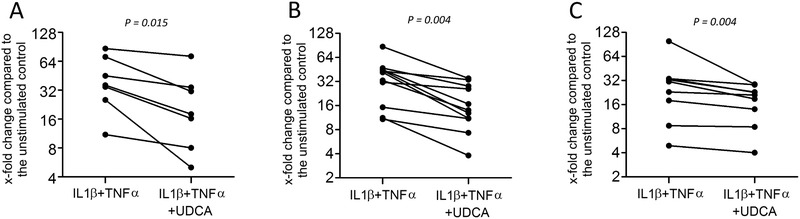
UDCA decreased the expression of proinflammatory cytokines in oral fibroblasts. Human gingival fibroblasts were exposed to IL1β and TNFα for 1 hour followed by the addition of UDCA for 3 hours. Relative gene expression for (**A**) IL1, (**B**) IL6, and (**C**) IL8 was determined by RT‐PCR. The dot blots show the data of independent experiments. Statistical analysis was based on Wilcoxon matched‐pairs signed rank test

All experiments were repeated at least three times. Graphs show the values of different experiments. Statistical analysis was based on Wilcoxon matched‐pairs signed rank test comparing two paired groups (Figs. 2, 4, and 5, Table 1) and Friedman test followed by Dunn's multiple comparisons (Fig. 1) of non‐parametric data and analysis was performed with using standard software.‖GraphPad Software, San Diego, CA.


**Table 1:** UDCA decreased production of IL6 in oral fibroblasts, HSC‐2, and RAW 264.7 cells

GF (pg/mL)	Basal	IL1β + TNFα	IL1β + TNFα + UDCA (*P* = 0.25)
Experiment 1	37.4	130.0	106.9
Experiment 2	38.2	166.1	88.6
Experiment 3	37.6	236.5	196.3

HSC‐2 (pg/mL)	Basal	IL1β + TNFα	IL1β + TNFα + UDCA (*P* = 0.25)
Experiment 1	29.1	115.7	53.8
Experiment 2	14.4	50.8	24.9
Experiment 3	21.9	69.1	29.2

RAW 264.7 (ng/mL)	Basal	Saliva	Saliva + UDCA (*P* = 0.25)
Experiment 1	0.1	29.1	27.5
Experiment 2	0.3	5.7	5.1
Experiment 3	0.3	19.4	15.2

## 3 RESULTS

### 3.1 UDCA decreased the viability of oral fibroblasts at millimolar concentrations

To evaluate the toxicity, gingival fibroblasts were exposed to various concentrations of UDCA. As indicated in Figure 1, gingival fibroblasts produced formazan crystals up to 500 µM, with a sharp decrease in viability at ≥1 mM. Based on these observations and in vitro research from others,^19^, ^20^ the following experiments on changes of the expression of inflammatory cytokines were performed at 100 µM UDCA. To rule out that the addition of the cytokines is an additional stressor, we exposed gingival fibroblasts, HSC‐2, and RAW 264.7 cells to IL1β and TNFα for the first two cell lines and saliva for the RAW cells followed by 100 µM UDCA. The presence of inflammatory cytokines in addition to UDCA does not significantly affect the formation of formazan crystals (see Supplemental Figure S1 in online *Journal of Periodontology*). Also, the cell membrane integrity of gingival fibroblasts was maintained by the combined use of 100 µM UDCA and inflammatory cytokines (see Supplemental Figure S2 in online *Journal of Periodontology*). Moreover, BCL2A1 being highly regulated by NFκB and exerts important pro‐survival functions, was strongly upregulated by IL1β and TNFα, but independent of UDCA (data not shown).^24^

### 3.2 UDCA decreased the expression of proinflammatory cytokines of oral fibroblasts

**Figure 3:**
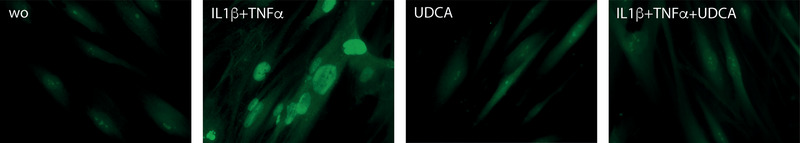
UDCA diminished the nuclear translocation of p65 in oral fibroblasts exposed to IL1β and TNFα. Human gingival fibroblasts were left untreated (wo; without) or exposed to IL1β and TNFα for 20 minutes followed by the addition of UDCA for 1 hour. Alexa 488 labeling appearing in green detected the NFkB p65 primary antibody. Note the nuclear translocation of the p65 subunit by the inflammatory cytokines and the concomitant suppression by UDCA. Pictures are taken at 20× magnification

The expression of inflammatory cytokines IL1, IL6, and IL8 was measured in oral fibroblasts, which were already exposed to the proinflammatory cytokines IL1β and TNFα. As expected, the treatment significantly increased the expression of three mentioned cytokines. There was a significant reduction in expression of IL1 (*P* = 0.015), IL6 (*P =* 0.004), and IL8 (*P* = 0.004) in presence of UDCA (Fig. 2). Immunoassay was applied to confirm the anti‐inflammatory capacity of UDCA based on the production of IL6 protein even though the level of significance was not reached based on a non‐parametric test (*P* = 0.25; Table 1). In support of these observations, UDCA reduced the nuclear translocation of p65 upon incubation with IL1β and TNFα (Fig. 3).

### 3.3 UDCA diminished the expression of proinflammatory cytokines of HSC‐2 cells

**Figure 4:**
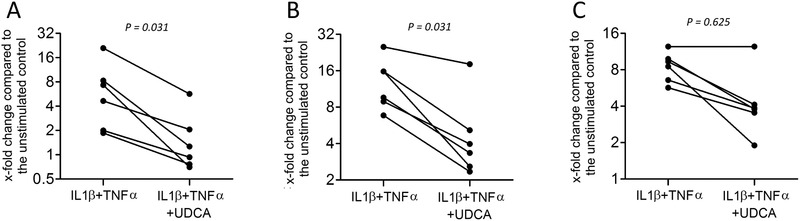
UDCA decreased the expression of proinflammatory cytokines of oral HSC‐2 cells. Human HSC‐2 cells were exposed to IL1β and TNFα for 1 hour followed by the addition of UDCA for 3 hours. Relative gene expression for (**A**) IL1, (**B**) IL6, and (**C**) IL8 was determined by RT‐PCR. The dot blots show the data of independent experiments. Statistical analysis was based on Wilcoxon matched‐pairs signed rank test

RT‐PCR showed that HSC‐2 cells pretreated with IL1β and TNFα also respond to UDCA with a significant decrease in expression of IL1 (*P* = 0.031) and IL6 (*P* = 0.031), as well as a moderate reduction in expression of IL8 (*P* = 0.625) (Fig. 4). Immunoassay data supported the reduction of IL6 release by HCS‐2 cells in presence of UDCA but failed to reach significance (*P* = 0.25; Table 1).

### 3.4 UDCA reduced the expression of proinflammatory cytokines of RAW 264.7 macrophages

**Figure 5:**
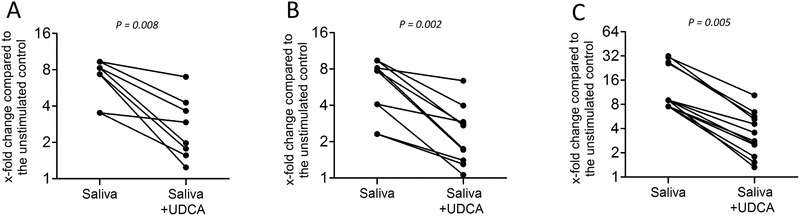
UDCA reduced the expression of proinflammatory cytokines of RAW 264.7 cells. Mouse RAW 264.7 macrophages were exposed to the human saliva for 1 hour followed by the addition of UDCA for 3 hours. Relative gene expression for (**A**) IL1α, (**B**) IL1β, and (**C**) IL8 was determined by RT‐PCR. The dot blots show the data of independent experiments. Statistical analysis was based on Wilcoxon matched‐pairs signed rank test

Considering that UDCA has anti‐inflammatory effects in lipopolysaccharide‐exposed RAW 264.7 cell,^17^ and saliva provokes an expression of proinflammatory cytokines in RAW 264.7 cells,^22^ it was undetermined if UDCA also reduces the saliva‐induced inflammatory reaction. We report here that UDCA significantly decreased the expression of IL1α (*P* = 0.008), IL1 β (*P* = 0.002), and IL6 (*P* = 0.005) in the presence of 5% saliva, making these observations particularly interesting for oral health research (Fig. 5). The reduction of IL6 production by UDCA was weak and did not reach significance (Table 1).

## 4 DISCUSSION

The scientific rational of this study was built upon the epidemiologic data indicating that patients with liver scars are at a higher risk for periodontal disease^2^, ^3^ and patients with liver cirrhosis are frequently treated with UDCA,^7^ a secondary bile acid with a potent anti‐inflammatory activity as reported for colitis models^9^, ^10^ but not for periodontitis models.^25^ Thus, there is a rational to believe that patients with liver cirrhosis may benefit from UDCA when they suffer from periodontitis. The present in vitro study is a proof‐of‐principle attempt aiming to confirm the anti‐inflammatory activity of UDCA in cells found in the periodontium. The main goal of the present study was therefore to show that UDCA exerts anti‐inflammatory properties using in vitro models of oral mesenchymal cells, oral epithelial cells, and macrophages.

If we relate our findings with those of others, it seems obvious that the anti‐inflammatory properties of UDCA are not restricted to the already reported lipopolysaccharide‐exposed 264.7 cells^17^ and other models of cytokine‐activated macrophages.^16^ We therefore extend existing evidence by showing that also saliva‐induced expression of proinflammatory cytokines is dampened by UDCA in 264.7 cells.^22^ Moreover, we present here pioneer data showing that UDCA exerts its anti‐inflammatory effects on the other cell lineages—the gingival fibroblasts and the oral human squamous carcinoma cell line HSC‐2. It should be mentioned that although IL6 decreased with UDCA in the supernatant from three independent experiments, the study was underpowered to reach the level of significance. Our data are also in line with observations that UDCA reduced the nuclear translocation of p65 in retinas from mice with diabetes.^26^ It thus seems clear that this anti‐inflammatory property is relevant in various cell types, including mesenchymal and epithelial cells that represent the periodontal tissue.

The clinical relevance of the present investigation remains at the level of a theory. The present data, however, provide a scientific rational to investigate the possible disease‐protecting role of UDCA in a periodontitis models, including those with ligatures, bacteria, or endotoxin injections.^27^ Moreover, it would be feasible to perform a longitudinal study on periodontal health in patients suffering from liver cirrhosis before and under UDCA therapy. Noticeably, systemic treatment of UDCA should be considered a complementary approach to regular oral hygiene and periodontal maintenance therapy. Taken together, the present research justifies investigations on the impact of UDCA on periodontal health in patients suffering from liver diseases.

The present study has limitations. First, a standard concentration of 100 µM UDCA was used in accordance with research on cardiomyocytes^19^ and CD4 T cells.^20^ Our concentration was slightly higher than the 40 µM UDCA used with mast cells,^21^ slightly lower than 200 µM UDCA in pancreatic cancer cells^18^ but considerably lower than the 1 mM UDCA of previous studies.^17^ In the present study, the 100 µM UDCA, alone and in the presence of inflammatory cytokines, were not cytotoxic in a series of viability assays performed with various cell types. Nevertheless, the pharmacologic profile of UDCA, particularly the concentration of UDCA in the periodontal tissues is unknown. Thus, it is not clear if our setting represents the clinical scenario. Second, periodontitis is a chronic inflammatory disease where poor oral hygiene causing plaque accumulation is considered a key pathologic factor. In vitro, a plaque‐based chronic inflammation cannot be simulated by simply exposing cells to saliva or inflammatory cytokines. Thus, it cannot be ruled out if the reported anti‐inflammatory action of UDCA is transient and not effective under plaque‐mediated chronic inflammation. Third, cell lines such as the human HSC‐2 tumor cells and the mouse 264.7 cells may not represent primary cells, suggesting that our data should be interpreted with caution. Finally, the molecular mechanisms responsible for the anti‐inflammatory action of UDCA are unclear and might involve the bile acid sensor farnesoid X receptor and a G‐protein‐coupled bile acid receptor, known as GPBAR1 or TGR5.^28^ Future studies should evaluate the systemic application of UDCA in mouse models of inflammatory osteolysis,^27^ with deficiency in the respective receptors.^29^

## 5 CONCLUSION

Our data suggest that UDCA can reduce the expression of proinflammatory cytokines of oral fibroblasts, oral human squamous carcinoma cells and macrophages, all of which are involved in the maintenance of periodontal health.

## AUTHOR CONTRIBUTIONS

Prof. Gruber and Dr. Talebian contributed to conception and design; contributed to acquisition, analysis, and interpretation; drafted the manuscript; critically revised the manuscript; gave final approval; and agreed to be accountable for all aspects of work. Dr. Panahipour contributed to acquisition, analysis, and interpretation; critically revised the manuscript; gave final approval; and agreed to be accountable for all aspects of work.

## Supporting information

Supplementary information

Supplementary information

Supplementary information
