# Supplementary material for: Ursodeoxycholic acid attenuates the expression of proinflammatory cytokines in periodontal cells
Source: J Periodontol. 2020 Feb 6;91(8):1098–104. doi: 10.1002/JPER.19-0013 (PMC7496100; doi:10.1002/JPER.19-0013)
Supplement: Supplementary file 1 — Supplementary information [file JPER-91-1098-s001.docx]

**Supplement Figure 1**

**
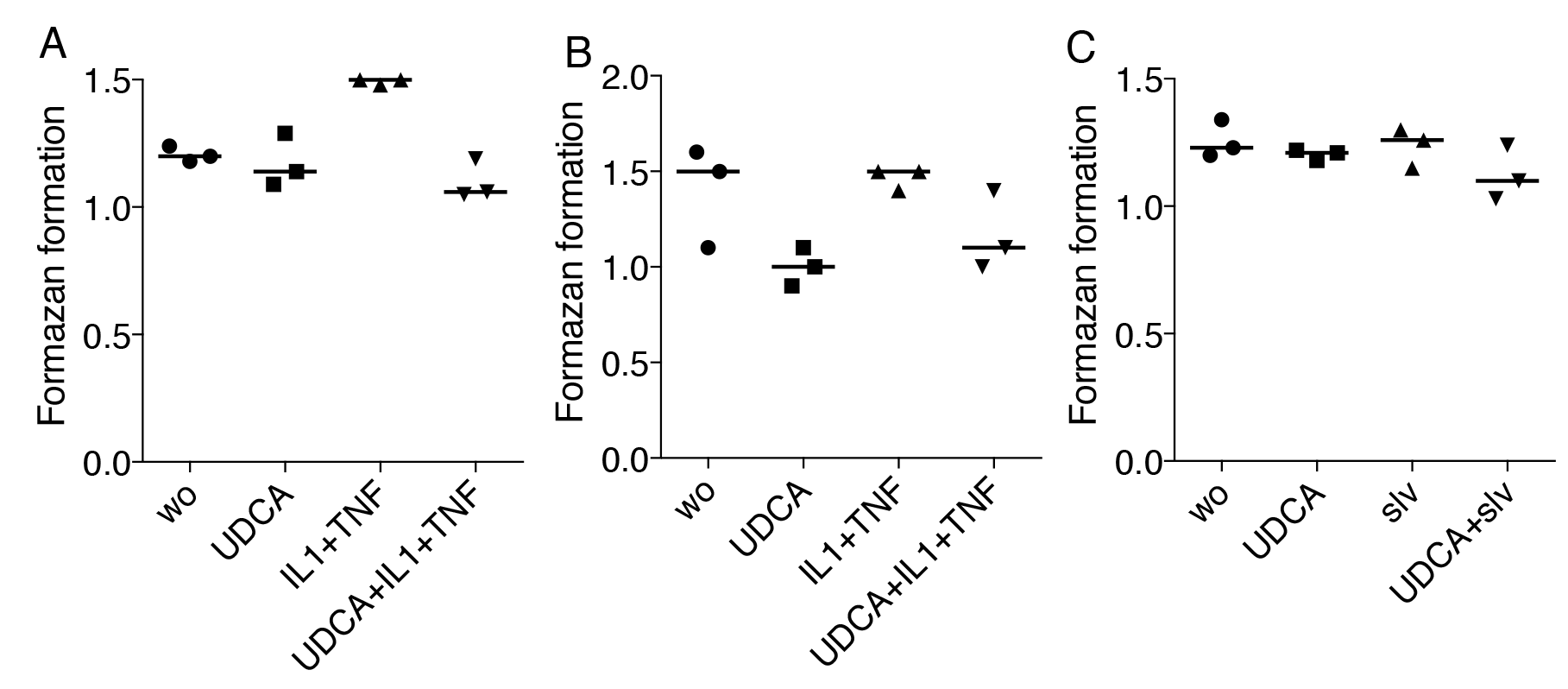
**

*Supplement Figure 1: Cell viability in the presence of UDCA in combination with cytokines*

Human gingival fibroblasts (A), HSC-2 cells (B) were stimulated with IL1β and TNFα at 5ng/ml for one hour and RAW 247.6 cells (C) were stimulated with 5% saliva (slv) for one hour, followed by the addition of UDCA at 100 µM in serum-free medium for 3 hours. Data show the formation of formazan crystals in optical density of three independent experiments. Based on Friedmann test, no post hoc analysis was performed; (A) p=0.054; (B) p=0.083; (C) p=0.61, respectively.
