# Supplementary material for: Ursodeoxycholic acid attenuates the expression of proinflammatory cytokines in periodontal cells
Source: J Periodontol. 2020 Feb 6;91(8):1098–104. doi: 10.1002/JPER.19-0013 (PMC7496100; doi:10.1002/JPER.19-0013)
Supplement: Supplementary file 2 — Supplementary information [file JPER-91-1098-s002.docx]

**Supplement Figure 2**


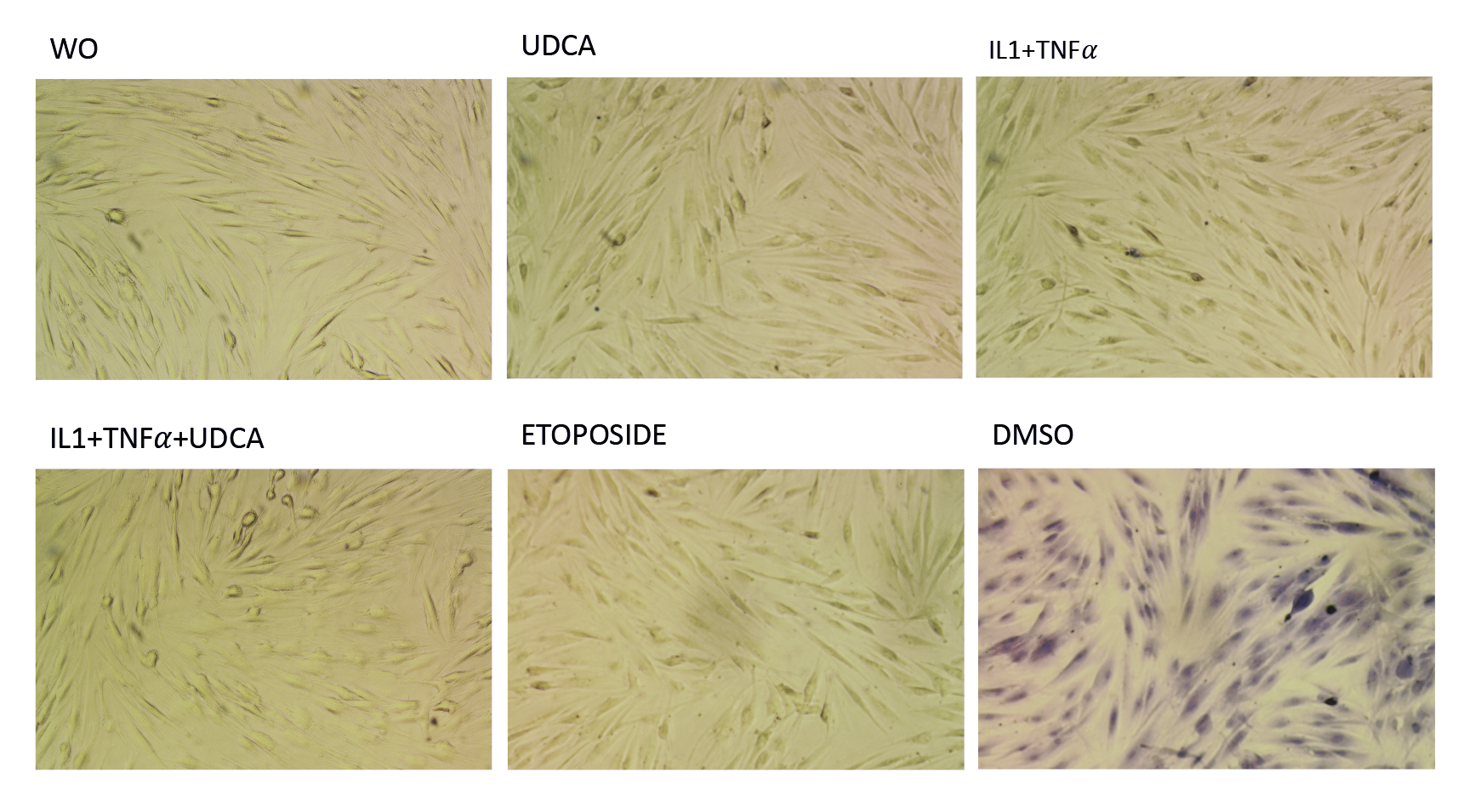


*Supplement Figure 2: Membrane integrity in the presence of UDCA in combination with cytokines*

Human gingival fibroblasts were stimulated with IL1β and TNFα at 5ng/ml for one hour followed by the addition of UDCA at 100 µM in serum-free medium for 3 hours. Cells were exposed to 0.4% trypan blue for one minute to determine the integrity of the cell membrane. Only concentrated DMSO impaired cell integrity indicated by the blue stain of the cytoplasm.
