# Supplementary material for: Ursodeoxycholic acid attenuates the expression of proinflammatory cytokines in periodontal cells
Source: J Periodontol. 2020 Feb 6;91(8):1098–104. doi: 10.1002/JPER.19-0013 (PMC7496100; doi:10.1002/JPER.19-0013)
Supplement: Supplementary file 3 — Supplementary information [file JPER-91-1098-s003.docx]

Table 1: The primers sequences for the cDNA preparation

Gene Forward Primer Sequence Reverse Primer Sequence

Human IL1β CTGATGGCCCTAAACAGATGAAGT AGCCCTTGCTGTAGTGGTGGT

Human IL6 GAAAGGAGACATGTAACAAGAGT GATTTTCACCAGGCAAGTCT

Human IL8 AACTTCTCCACAACCCTCTG TTGGCAGCCTTCCTGATTTC

Human BCL2A1 CAGGAGAATGGATAAGGCAAA CCAGCCAGATTTAGGTTCAAA

Mouse IL1α TTGGTTAAATGACCTGCAACA GAGCGCTCACGAACAGTTG

Mouse IL1β AAGGGCTGCTTCCAAACCTTTGAC ATACTGCCTGCCTGAAGCTCTTGT

Mouse IL6 GCTACCAAACTGGATATAATCAGGA CCAGGTAGCTATGGTACTCCAGAA
